# Patient-Derived iPSCs Reveal Evidence of Telomere Instability and DNA Repair Deficiency in Coats Plus Syndrome

**DOI:** 10.3390/genes13081395

**Published:** 2022-08-05

**Authors:** Noufissa Oudrhiri, Radhia M’kacher, Diana Chaker, Bruno Colicchio, Claire Borie, Eric Jeandidier, Alain Dieterlen, Frank Griscelli, Annelise Bennaceur-Griscelli, Ali G. Turhan

**Affiliations:** 1 Inserm UMR_S_1310, 94805 Villejuif, France; 2 APHP Paris Saclay Service Onco-Hematology and Cytogenetics, Paul-Brousse, 94805 Villejuif, France; 3 INGESTEM National iPSC Infrastructure, 94805 Villejuif, France; 4 Centre for iPSC Therapies (CITHERA) INSERM UMS 45, Genopole, 91000 Evry-Courcouronnes, France; 5 IRIMAS, Institut de Recherche en Informatique, Mathématiques, Automatique et Signal, Université de Haute-Alsace, 68070 Mulhouse, France; 6 Laboratoire de Génétique, Groupe Hospitalier de la Région de Mulhouse Sud-Alsace, 68070 Mulhouse, France; 7 Département de Biologie Médicale et Pathologie Médicales, Service de Microbiologie, Gustave Roussy Cancer Campus, 94805 Villejuif, France; 8 Faculté des Sciences Pharmaceutiques et Biologiques, Université Paris Descartes, Sorbonne Paris Cité, 75006 Paris, France; 9 APHP-Paris Saclay Service d’Hématologie-Bicetre, 94270 Le Kremlin Bicêtre, France

**Keywords:** Coats plus syndrome, telomere, telomerase, alternative lengthening of telomere (ALT), CTC1, chromosomal instability

## Abstract

Coats plus (CP) syndrome is an inherited autosomal recessive condition that results from mutations in the conserved telomere maintenance component 1 gene (*CTC1*). The CTC1 protein functions as a part of the CST protein complex, a protein heterotrimer consisting of CTC1–STN1–TEN1 which promotes telomere DNA synthesis and inhibits telomerase-mediated telomere elongation. However, it is unclear how *CTC1* mutations may have an effect on telomere structure and function. For that purpose, we established the very first induced pluripotent stem cell lines (iPSCs) from a compound heterozygous patient with CP carrying deleterious mutations in both alleles of *CTC1*. Telomere dysfunction and chromosomal instability were assessed in both circulating lymphocytes and iPSCs from the patient and from healthy controls of similar age. The circulating lymphocytes and iPSCs from the CP patient were characterized by their higher telomere length heterogeneity and telomere aberrations compared to those in control cells from healthy donors. Moreover, in contrast to iPSCs from healthy controls, the high levels of telomerase were associated with activation of the alternative lengthening of telomere (ALT) pathway in CP-iPSCs. This was accompanied by inappropriate activation of the DNA repair proteins γH2AX, 53BP1, and ATM, as well as with accumulation of DNA damage, micronuclei, and anaphase bridges. CP-iPSCs presented features of cellular senescence and increased radiation sensitivity. Clonal dicentric chromosomes were identified only in CP-iPSCs after exposure to radiation, thus mirroring the role of telomere dysfunction in their formation. These data demonstrate that iPSCs derived from CP patients can be used as a model system for molecular studies of the CP syndrome and underscores the complexity of telomere dysfunction associated with the defect of DNA repair machinery in the CP syndrome.

## 1. Introduction

Telomeres are repeated DNA sequences *(T2AG3)n* associated with a protein complex termed shelterin at the end of chromosomes [1]. Telomeres play a central role in the genome stability and integrity by protecting chromosomes from abnormally sticking together or degrading [2,3]. Telomere dysfunction is associated with activation of the DNA damage response machinery resulting in chromosome end-to-end fusions and instability [4,5]. During the past years, the implication of telomeres in the pathogenesis and progression of several diseases has been extensively demonstrated [1]. Disturbed telomere homeostasis plays a major role several human diseases. One such disease is the Coats plus syndrome (CP) that presents with cerebro-retinal microangiopathy with calcifications and cysts (CRMCC). CP is classified as a telomeropathy syndrome caused by deleterious recessive mutations in *CTC1* [6]. The *CTC1* gene (OMIM ≠ 613129), located on chromosome 17p13.1, encodes a 1217 amino-acid nuclear protein. CTC1 is part of the multiprotein complex CTC1–STN1–TEN1 (CST) that is a conserved single-stranded DNA-binding protein complex promoting telomere DNA synthesis and inhibiting telomerase-mediated telomere elongation [7,8].

Coats syndrome is a highly pleiotropic disorder particularly affecting the eye, brain, bone, and gastrointestinal tract. The clinical diagnosis of CP is suggested in the presence of a combination of bilateral retinopathy, intracranial calcifications, leukodystrophy, anemia, osteopenia, and poor bone healing [8]. Coats syndrome patients present clinical features distinct from other telomere diseases such as congenital dyskeratosis [9]. In experimental models of CP, *CTC1*-null mice display rapid telomere shortening, accompanied by global proliferative defects and premature death due to bone marrow failure [10]. *CTC1* mutations identified in a CP patient resulted in telomere dysfunction and a severe reduction in proliferation, suggesting that the pathogenesis of CP may be caused by failure of cell proliferation due to compromised maintenance of telomeres [11,12]. Mechanisms underlying telomere dysfunction and telomere maintenance, as well as the associated chromosomal instability in CP syndrome, are not yet understood.

The technology of reprogramming somatic cells into induced pluripotent stem cells (iPSCs), offers an interesting approach to analyze telomere dysfunction, DNA repair defect, and chromosomal instability in telomeropathies. According to their pluripotent phenotype including activation of *hTERT* expression, as well as their ability to differentiate into any cell lineage, iPSCs may be used as tools to investigate telomere defects, as well as cellular proliferation failures in patients with CP syndrome [13].

Here, we report the establishment of a patient-derived iPSC model of CP. Peripheral blood monocytes of a CP patient presenting germline biallelic deleterious *CTC1* mutations were reprogrammed into iPSCs. We investigated telomere dysfunction and maintenance in CP-iPSCs, as well as in differentiated cells derived from these iPCSs. We show that, in comparison with cells of healthy controls, CP-iPSCs and their derivative mesenchymal stem cells (MSC) express higher levels of telomerase and harbor telomere dysfunction, altered nuclear architecture, and higher radiation sensitivity with activation of the DNA damage response and premature senescence. We also demonstrate that mechanisms of alternative lengthening of telomeres (ALT) contribute to the maintenance of telomeres, in addition to a high level of hTERT expression. Thus, our CP-iPSCs constitute a novel model system for studying molecular mechanisms underlying CP disease initiation and progression, as well as for developing novel therapeutic approaches.

## 2. Materials and Methods 

### 2.1. iPSC Line and Derivative Cells

Peripheral blood samples were obtained, with informed consent according to the Declaration of Helsinki and the local ethics committee, from a patient with Coats plus disease, confirmed by a germline deleterious mutation in both alleles of the CTC1 gene [7]. The iPSC line was generated from peripheral blood mononuclear cells (PBMCs) according to our previously published protocol [14].

Briefly, PBMCs were cultured for 4 days in Myelocult medium (Stem Cell Technologies, Saint-Egrève, France) in the presence of hSCF (100 ng/mL), hFLT-3 (100 ng/mL), hIL-6 (20 ng/mL), hIL-3 (20 ng/mL), and hIL7 (20 ng/mL) (Peprotech). At day + 4 of the culture, 2 × 10^5^ mononuclear cells were transduced overnight with Sendai virus containing Oct3/4, Sox2, Klf4, and c-Myc (CytoTune^®^-iPS Sendai Reprogramming Kit, Life technologies, Saint-Aubin, France), at a multiplicity of infection (MOI) of 15. Transduced cells were cultured in the presence of bFGF (10 ng/mL) in Mitomycin C-treated murine embryonic fibroblast (MEF) feeders. Colonies appearing after 2–3 weeks were picked, and individual clones were characterized, expanded, and cryopreserved.

CP and control iPSC clones were characterized using pluripotency markers by FACS (SSEA-3, SSEA-4, and Tra1-60 staining) and teratoma formation assays in NSG mice, revealing evidence of differentiation toward tissues belonging to three germ layers (endoderm, ectoderm, mesoderm).

iPSC lines were expanded on mitomycin C (20 mg/mL)-inactivated mouse embryonic fibroblasts in Knock-Out DMEM medium supplemented with 20% Knock-Out Serum Replacement, 1 mM l-glutamine, 100 mM β-mercaptoethanol, 0.5% penicillin/streptomycin (Invitrogen, Carlsbad, CA, USA), and 5 ng/mL bFGF (Miltenyi Biotech, Bergisch Gladbach, Germany). Half of the medium was changed daily, and cells were passaged weekly with collagenase type IV (Invitrogen). Cultures were incubated in a humidified atmosphere at 37 °C, 5% CO_2_. After 25 passages, a pool of colonies was recovered and expanded in feeder-free conditions on 1% Geltrex™ matrix (1 mL in DMEM/F12 for a 60 mm plate), before incubating for 1 h at 37 °C using Essential 8 medium (Life-technologies, Pays) for further characterization. 

Mesenchymal stem cells (MSCs) were induced by culturing iPSCs from the CP patient and from healthy controls (passage 30) in DMEM/F12 (Invitrogen) medium, supplemented with 10% heat-inactivated FBS (Hyclone), 1 ng/mL b-FGF, 0.1 mM nonessential amino acids, 1 mM l-glutamine, 0.1 mM β-mercaptoethanol, and 1× penicillin/streptomycin (all from Invitrogen). MSCs were characterized by flow cytometry on the basis of their loss of pluripotent marker expression (Oct4, Sox2, and Nanog), the expression of CD90, CD105, CD146, CD54 (ICAM-1), CD73, and HLA class I (HLA-ABC) and class II (HLA-DR), and the lack of CD34 and of CD45 expression. Multipotency of the MSCs was validated by their differentiation along the osteogenic, chondrogenic, and adipogenic lineages. Immunocytochemistry was used with polyclonal goat anti-mouse FABP-4, monoclonal goat antihuman Aggrecan, and mouse anti-human Osteocalcin antibodies.

### 2.2. Preparation of Metaphase Spreads

Peripheral blood lymphocytes, iPSCs, and their derivative cells were exposed to colcemid (0.1 µg/mL) (Gibco KaryoMAX,) for 2 h at 37 °C, 5% CO_2_, in a humidified atmosphere to arrest dividing cells in metaphase. After harvesting the cells, they were centrifuged for 7 min at 1400 rpm at room temperature, the supernatant was removed, and the cell pellet was resuspended in a solution of warm (37 °C) 0.075 M potassium chloride (KCl) (Merck, NJ, USA) and incubated for 20 min in a 37 °C water bath (hypotonic shock). The cells were prefixed by adding approximately five drops of fixative (ethanol/acetic acid, 3:1 *v*/*v*) to each tube under agitation, and the tubes were centrifuged for 7 min at 1400 RPM at room temperature. The supernatant was removed, and cells were resuspended in the fixative solution followed by centrifugation using the same parameters. After two additional rounds of these fixation steps, the cells were stored in the fixative solution at 4 °C overnight and the metaphases spread on cold wet slides the next day. The slides were dried overnight at room temperature and stored at −20 °C until further use [15].

### 2.3. Telomere–Centromere Staining Followed by M-FISH Technique (TC + M-FISH)

Telomeres and centromeres were hybridized with a Cy-3-labeled PNA probe specific for TTAGGG for telomeres and an FITC-labeled probe specific for centromere sequences (Eurogentec, Liège, Belgium), as previously described [16,17]. Briefly, slides were washed with 1× PBS and fixed with 4% formaldehyde at room temperature. After rinsing three times (3 × 5 min) with PBS, they were treated with pepsin (0.5 mg/mL) at 37 °C for 5 min. After rinsing three times (3 × 5 min) with PBS, the slides were sequentially dehydrated with 50%, 70%, and 100% ethanol and air-dried. The telomere and centromere probes were added to the slides and subsequently denatured on a hot plate at 80 °C for 3 min and then incubated in the dark for 1 h at room temperature. The slides were subsequently rinsed with 70% formamide/10 mM Tris pH 7.2 two times for 15 min (for 2 × 15 min) and then in 50 mM Tris pH 7.2/150 mM NaCl/0.05% Tween-20 (3 × 5 min). After a final rinse in PBS, the slides were counterstained with DAPI and mounted in PPD After telomere quantification and the automatic capture of metaphases with telomere and centromere staining, the slides were washed with 2× SCC for 30 min at 70 °C. After rinsing with 0.1× SSC for 1 min at room temperature, they were denatured using NaOH, subsequently washed (1 min) with 0.1× SCC and 2× SSC, sequentially dehydrated in 70%, 95%, and 100% ethanol, and air-dried. After denaturation of the M-FISH probe (M-FISH 24XCyte, MetaSystems, Altlussheim, Germany) for 5 min at 75 °C, the probe was added to the slides, and the slides were incubated at 37 °C for 2 days. The slides were subsequently rinsed with 0.4× SSC for 2 min at 72 °C and then in 2× SSC/0.005% Tween-20. The slides were counterstained with DAPI and mounted in PPD [16,18].

### 2.4. Telomere Quantification

Quantitative image acquisition was performed using MetaCyte software (MetaSystems, version 3.9.1, Altlussheim, Germany). The exposure and gain settings remained constant between captures. The analysis of telomere signals was performed using TeloScore Software (Cell Environment, Paris, France). The mean fluorescence intensity (FI) of telomeres was automatically quantified and analyzed in 10,000 nuclei on each slide. The experiments were performed on triplicate slides.

Telomere length, measured as the mean fluorescence intensity (FI), strongly correlated with telomere length measured by conventional Southern blot analysis using the telomeric restriction fragment (TRF) (*R*^2^ = 0.721 and *p* = 2.128 × 10^−8^). The mean telomere length is expressed in kb.

### 2.5. Scoring of Telomeres and Chromosomal Aberrations

The images of an average of 100 metaphases were captured using automated acquisition module Autocapt software (MetaSystems, version 3.9.1, Altlussheim, Germany) and a ZEISS Plan-Apochromat 63×/1.40× oil and CoolCube 1 Digital High-Resolution CCD Camera with constant settings for exposure and gain.

Telomere aberrations were assessed using the following criteria: (*i*) telomere loss was defined as a signal-free end at a single chromatid, an aberration that leads to telomere-end-fusion and breakage/fusion/bridge cycles [19]; (*ii*) telomere doublets or telomere fragility were defined as more than one telomere signal at a single arm, an aberration signaling inadequate telomere replication, and the dysfunction of shelterin proteins [20,21]; (*iii*) telomere deletion was defined as the loss of telomere signals on both arms of either the p or q arms, an aberration considered to represent double-strand breaks, leading to the activation of DNA repair mechanisms. Automatic scoring of these aberrations was performed using ChromoScore software (Cell Environment, Paris, France), and an operator validated and excluded the false aberrations.

### 2.6. Micronucleus Assay

For these experiments, we avoided using cytochalasin B due to its cell toxicity [22]. Cells were incubated in a humidified atmosphere of 5% (*v*/*v*) CO_2_ in air at 37 °C until confluency, and cells spread by trypsinization. The culture medium was discarded, and hypotonic shock was induced by incubating the cells with 18 mM KCl at room temperature. The cells were then fixed with acetic acid/(methanol (1:3, *v*/*v*), and the cell suspension was dropped onto the slides. The slides were stored at −20 °C until further use [23].

### 2.7. In Vitro Irradiation

iPSC and MSC were irradiated at various doses (0.1, 1, 2, and 4 Gy) using X-RAD 320 with a dose rate of 1 Gy/min at room temperature. Cells were maintained at 37 °C, and survival was scored after 3 weeks. Second irradiation was performed for proliferative cells after the first irradiation. Cell survival and radio-induced chromosomal aberrations were analyzed 24 h and 1 month, respectively, after irradiation.

### 2.8. Immunofluorescence and Immunofluorecent FISH (IF-FISH) 

Cells were cytospun onto poly-l-lysine-coated glass slides at 700 rpm for 4 min, fixed with 10% formalin for 10 min, and treated with 0.25% Triton X-100 solution for 10 min. After blocking with 5% bovine serum albumin (Sigma Aldrich, Saint-Quentin-Fallavier, France), the cells were incubated overnight in 4 °C with primary antibody. After washing in PBS, cells were treated with cyanine 3-labeled anti-mouse IgG (Invitrogen, Carlsbad, CA, USA) or FITC-labeled anti-rabbit (Sigma Aldrich, Saint-Quentin-Fallavier, France) secondary antibody at 37 °C for 45 min. Cells were mounted in *p*-phenylene diamine after counterstaining with 4,6-diamidino-2-phenylindole (Sigma Aldrich, Saint-Quentin-Fallavier, France). As a negative control, staining was carried out in the absence of primary antibody [17]. γH2AX, 53BP1, and ATM antibodies were used as DNA repair proteins, and hTERT, PML, and TRF2 antibodies were used for telomere maintenance.

IF-FISH was performed using a protocol similar to one described previously [24]. The suspension of cells (200 μL at concentration of 60,000 cells/mL) was subsequently applied to polylysine slides by cytospin. Following fixation (paraformaldehyde (PFA) 3%, sucrose 2%), cells were immunostained as described. Prior to telomere hybridization with the PNA probe (CCCTAA)3-FITC, cells were successively fixed (PFA 4%, 2 min), washed in PBS, and dehydrated (50/70/100 ethanol).

### 2.9. Senescence β-Galactosidase Cell Staining

To assess senescent cells the Senescence β-Galactosidase staining kit (Cell signaling technology, Pays) was used according to the manufacturer’s instructions. Briefly, cytochemical detection of β-galactosidase (SA-βgal) activity was detected by incubating in situ fixed cells with the chromogenic β-gal substrate X-gal. A blue color developed in some of the cells within 2 h, but staining was maximal after 12–16 h. After staining, the cells were washed with phosphate-buffered saline (PBS) and viewed by bright-field or phase-contrast microscopy. Alternatively, the fixed and stained cells were rinsed with methanol and air-dried prior to microscopic inspection. The blue cells positive for SA-βgal activity were counted among the total cell population in the field of view. 

### 2.10. Statistical Analysis

Data were expressed as the mean ± standard deviation and analyzed using the Wilcoxon–Mann–Whitney rank sum test (comparison of two subgroups) or the Kruskal–Wallis nonparametric test (comparison of three subgroups). A *p*-value <0.05 was considered statistically significant. All statistical analyses were conducted with the R software (ggpubr and plotROC libraries) (R package version 0.4.0 2020).

## 3. Results

### 3.1. Telomere Heterogeneity and Aberrations and Chromosomal Instability in Peripheral Blood Lymphocytes of a CP Patient

The clinical history of the patient was previously reported [7]. Briefly, an 18 year old male patient was admitted in the hospital for repeated seizures. Cerebral imaging disclosed asymmetrical leukopathy, intracranial calcification, and cysts. According to his anamnesis, he also had an intrauterine growth retardation, skeletal demineralization and osteopenia, and bilateral exudative vitreo-retinopathy reminiscent of Coats plus syndrome. Recurrent gastrointestinal hemorrhages caused by telangiectatic and angiodysplastic changes were evident. Blood transfusions were required as a result of his extensive gastrointestinal (GI) bleeding. Genetic analysis revealed that he was compound heterozygous with deleterious mutations in exon 6 of *CTC1*, c.833G > T/p.G278V and c.841T > C/p.Y281H. Each of the variant alleles was inherited from his parents.

**Figure 1:**
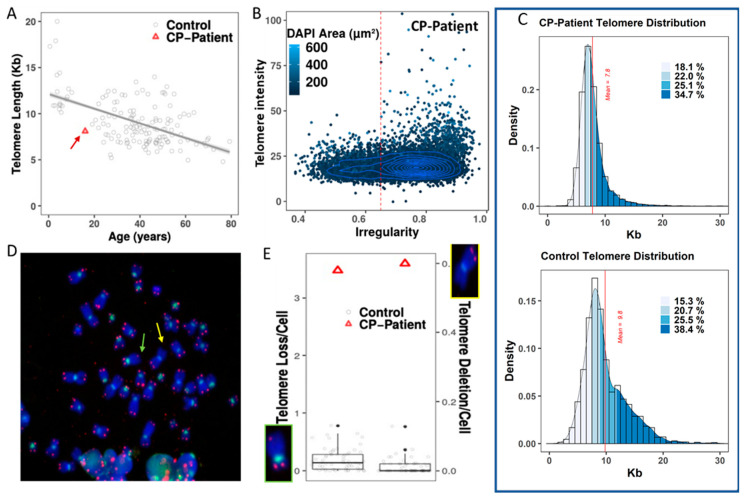
Telomere quantification and telomere aberrations in peripheral blood lymphocytes in CP-patient. (**A**) The telomere length of healthy donors is age-dependent. The regression line indicates telomere shortening with age in healthy donors (79 bp per year; *Y* = 12.1 − 0.79*X*; *R*^2^ = 0.29). Coats plus patient cells (red triangle with red arrow) have short telomeres compared to control cells (black circle). (**B**) Higher irregularity of nuclei of the CP peripheral lymphocytes. (**C**) Shorter telomere length and more narrow distribution of heterogeneity in peripheral blood lymphocytes of CP patient than in control cells. (**D**) FISH on metaphase from CP patient with probes specific for telomere (red) and centromere (green) sequences, respectively. The photo depicts a high frequency of telomere loss (green arrow) and telomere (63× magnification) deletion (yellow arrow). (**E**) Significant high rate of telomere loss (left insert and left panel) and of telomere deletion (right insert and right panel) in peripheral blood lymphocytes of CP patient (red triangle) compared to healthy controls (black circle).

First, we used FISH with telomere-specific probes to assess telomeres in peripheral blood lymphocytes of the CP patient and of healthy donors. The signals were automatically acquired, and telomere fluorescence intensity was quantified in 10,000 cells (Figure 1A). In CP-patient cells, telomeres were significantly shorter than those in cells of healthy controls with similar age (7.8 kb for CP-patient; 9.8 for control; *p* < 10^−4^) (Figure 1A). Although high intercellular heterogeneity in telomere lengths was observed, no critically short telomeres were detected (Figure 1B,C). For detailed inspection of telomeres on each chromosome, FISH with telomere- and centromere-specific probes was applied on metaphases from peripheral lymphocytes of the patient and of healthy controls. Quantification of the telomere signals confirmed the high intercellular heterogeneity in fluorescence intensity, as well as between individual chromosomes (Figure 1D). Interestingly, a high frequency of telomere loss and telomere deletion (for definition, cf. Section 2.5) was found in patient chromosomes compared to those in healthy donors (CP patient: 3.5 loss and 0.6 deletion per cell; control: 0.22 and 0.03, respectively; *p* < 10^−5^ and *p* = 0.04, respectively) (Figure 1E). Thus, these data demonstrate that peripheral blood lymphocytes of the CP patient displayed an abnormal telomeric phenotype. Moreover, telomere dysfunction in this CP patient was associated with an abnormal and irregular nuclear morphology (Figure 1B). 

**Figure 2:**
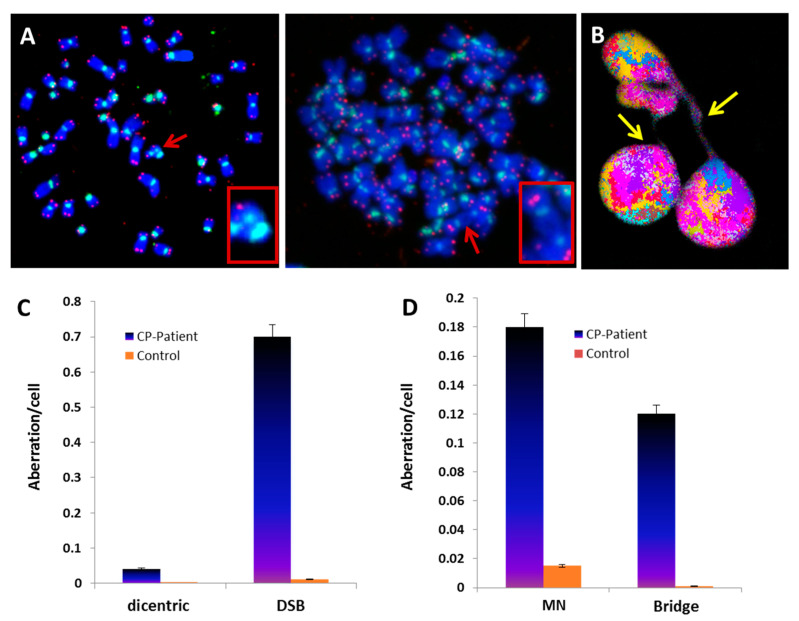
Chromosomal instability detected in circulating lymphocytes of CP patient. (**A**) FISH on metaphases from CP patient hybridized with telomere (red) and centromere-specific (green) probes. The photos depict dicentric chromosomes (red arrow and insert) in diploid and aneuploid metaphases (63× magnification). (**B**) Morphological alterations of CP patient nuclei with a large anaphase bridge related to a dicentric chromosome caused by chromosome end-to-end fusion (63× magnification) (yellow arrow). (**C**) High rate of dicentric chromosomes and DSBs resulting from all scored aberrations including telomere deletion considered as a DSB. (**D**) Higher rate of micronucleus (MN) and anaphase bridge formation in CP patient cells compared to those of healthy controls.

Telomere loss can lead to chromosome end-to-end fusions resulting in the formation of dicentric chromosomes [25]. Such chromosome aberrations are considered a superior biomarker of chromosomal instability. A significantly higher frequency of dicentric chromosomes was observed in circulating lymphocytes of the CP patient (Figure 2A) compared to that in healthy donors (Figure 2B) (*p* < 10^−8^). The difference was even larger when we calculated the total number of double-strand breaks (DSB) required for generating the recorded chromosome aberrations (*p* < 10^−6^ (Figure 2C)).

Next, we analyzed circulating lymphocytes of the CP patient for the presence of anaphase bridges that are a consequence of dicentric chromosome formation, as well as the frequency of micronuclei (Figure 2B). Circulating lymphocytes of the CP patient displayed an increased proportion of anaphase bridges compared to controls (0.12 anaphase bridge/cell in CP patient cells vs. 0.001 in control cells, *p* < 10^−6^). Furthermore, the fraction of cells with a lagging chromosome or an acentric chromosome in the form of micronuclei (MN) differed significantly between CP patients and controls (0.18 MN in CP patient cells vs. 0.015 in control cells, *p* < 10^−6^) (Figure 2D). Together, these data demonstrate that CP patient lymphocytes displayed telomere dysfunction associated with chromosomal instability.

### 3.2. Establishment of Coats Plus Induced Pluripotent Stem Cell Line

Telomerase activity is extinguished in most somatic cells during embryonic differentiation, but it remains active in some tissues such as male germ cells, activated lymphocytes, and certain types of adult stem-cell populations [26]. Thus, to study the effect of mutant *CTC1* proteins on the function of CST complexes and their interaction with telomerase in a patient-specific CP disease model, an easily accessible and reproducible source of telomerase-expressing cells is required.

Therefore, we aimed at establishing stable patient cell lines by reprogramming PBMCs into iPSCs with nonintegrative viral transduction vectors of Oct3/4, Sox2, Klf4, and cMyc [27]. Such stem cells have the advantage of expressing telomerase constitutively and to differentiate into various cell lineages that are affected by the pleiotropic *CTC1* variants of CP patients. 

The iPSC colonies showed the characteristic morphology of iPSCs on mouse embryonic fibroblast feeders (MEF) and expressed the typical markers of pluripotency: Nanog, Sox2, SSEA4, and TRA-1-60. Genomic sequencing of iPSC colonies confirmed the presence of the patient’s *CTC1* mutations that were originally identified in his PBMCs [7]. Moreover, mesenchymal stem cells (MSC) could be generated from the iPSCs and their multipotency documented by the expression of osteogenic, chondrogenic, and adipose lineage markers.

### 3.3. Evaluation of the Involvement of Telomerase and/or ALT Pathway for Telomere Maintenance in CP Cells 

**Figure 3:**
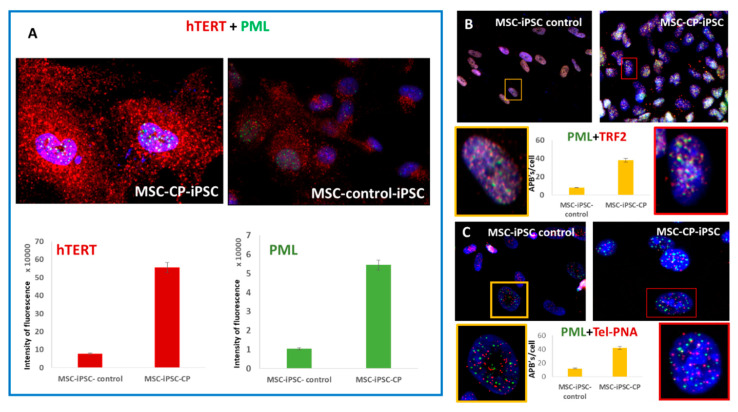
Telomerase and ALT pathway in MSCs derived from CP and control iPSCs. (**A**) Significantly higher expression of hTERT (red signal) and ALT proteins (green signal) in MSC-CP-iPSCs compared to MSC-control-iPSCs. (**B**) Colocalization of PML bodies and TRF2 protein (red signal) in MSC-CP-iPSCs. (**C**) Similarly, APBs were largely observed after immunofluorescence staining of PML and PNA telomere FISH staining (red signal) (IF-FISH) in only MSC-CP-iPSCs compared to MSC-control-iPSCs (63× magnification).

We previously demonstrated a strong correlation between telomerase activity and hTERT expression [17]. First, we indirectly explored the telomerase activity through the detection of hTERT protein amount in CP-iPSCs and MSCs by immunofluorescence. A significant increase in hTERT expression was detected in CP-iPSCs in comparison with their parental cells before reprogramming. hTERT expression was twofold higher in CP-iPSC-derived MSCs as compared to their normal iPSC-derived counterparts (Figure 3A). Nevertheless, the heterogeneity of telomere length with the presence of very long telomeres identified by Q-FISH (Figure 1) suggests that ALT mechanisms are also active in MSC derived from CP-iPS cells. Therefore, we analyzed markers for ALT using colocalization of PML protein with telomeres/telomeric proteins to identify ALT-associated PML bodies (APBs) [28,29]. High numbers of PML bodies were detected in MSC from CP-iPSCs compared to MSCs from healthy iPSCs (Figure 3A). To identify APBs, the colocalization of PML and telomeres via TRF2 or PML/PNA telomeres (IF-FISH) was scored. The distribution of the number of PML-TRF2 foci is shown in Figure 3B, as along with the identification of PML/PNA telomeres (Figure 3C). MSC from CP-iPSCs exhibited a higher frequency of APBs compared to MSCs from control iPSCs (Figure 3B,C). Overall, these data demonstrate the coexistence of telomerase upregulation by hTERT expression probably related to the CTC1 mutation, as well as the activation of ALT in MSC from CP-iPS cells.

### 3.4. Aberrant Telomere Elongation and Chromosomal Instability in CP-iPSCs and Derivative Cells 

**Figure 4:**
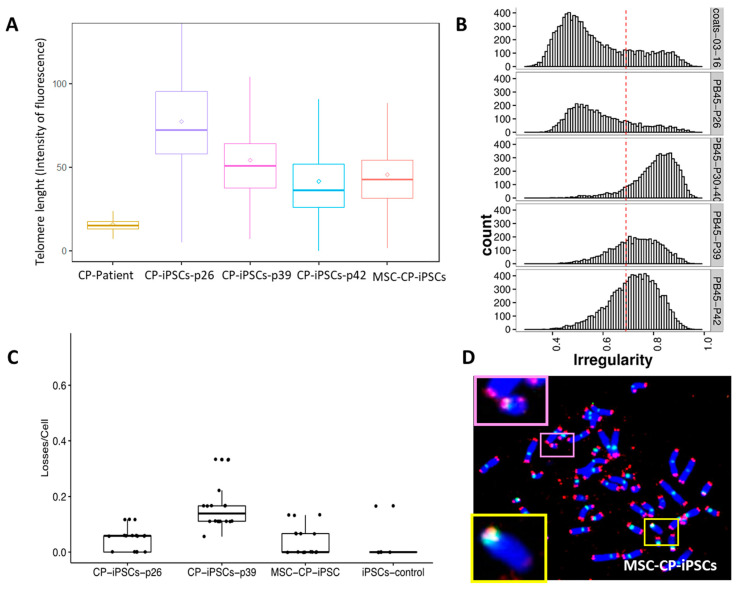
Telomere length and telomere aberration in CP-iPSCs. (**A**) Telomere length of peripheral blood lymphocytes of the CP patient, of CP-iPSCs at passages 26, 39, and 42, and of CP-iPSC-derived MSCs. (**B**) The decrease in telomere length in iPSCs was associated with the morphological modifications of nuclei such as their area and irregularity. (**C**) Telomere loss scored in iPSCs at different passages and in their derived MSCs showing a higher rate of telomere loss in CP-iPSCs compared to iPSCs from healthy donors. (**D**) Metaphase from MSC-CP-iPSC after telomere (red signal) and centromere staining (green signal) showing a high rate of telomere loss (upper insert) and deletion (lower insert) (63× magnification).

Telomere length was measured in CP-iPSCs during iterative passages. In CP-iPSCs, telomeres were elongated compared to those of circulating CP-lymphocytes. However, telomere attrition was observed during successive passages, with a significant and progressive shortening of telomere length between early (P26) and later passages (P39, P42) (Figure 4A). 

This decrease in mean telomere length in CP-iPSCs coincided with their accentuated telomere heterogeneity and their abnormal nuclear morphology (Figure 4B). Telomere lengths of MSC-CP-iPSCs were significantly shorter than telomere lengths of their parental CP-iPSCs (Figure 4A). 

To further determine the telomere phenotype of CP-iPSCs, we examined telomere aberrations, i.e., signs of telomere instability such as sister telomere loss or terminal deletion of telomeres on both sister chromatids of the p or q arms (Figure 4C). Analysis of telomeric FISH signals on metaphase spreads revealed a significantly high rate of telomere loss and deletions in CP-iPSCs and in MSC-CP-iPSCs compared to iPSCs derived from healthy donors (Figure 4C).

**Figure 5:**
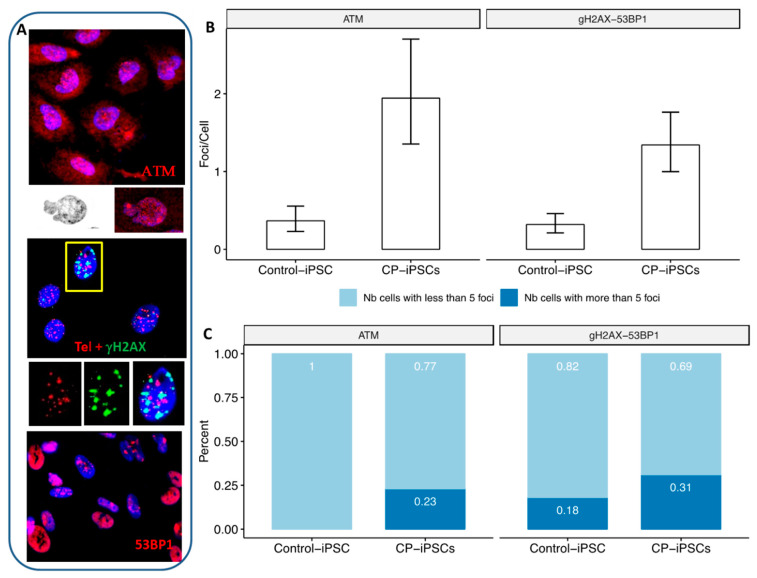
Expression of DNA repair proteins in CP-iPSCs. (**A**) CP-iPSCs cells after immunostaining of phos-ATM (red signal), 53BP1 (red signal) and γH2AX (green signal) showing the presence of multiple foci colocalized with telomere sequences (red signal) (40× magnification). (**B**) Foci rate of phos-ATM expression and γH2AX/53BP1 proteins in CP-iPSCs compared to control iPSCs showing a significant higher rate in CP-iPSCs. (**C**) Significant difference between the frequencies of CP-iPSCs with higher rate of ATM and γH2AX/53BP1 (more than five foci) compared to control iPSCs.

Telomere deletions may be considered as DSBs, leading to activation of a DNA repair pathway. Increased numbers of γH2AX and 53BP1 foci, biomarkers of DSBs, associated with the activation of ATM, were present in CP-iPSCs compared to those in healthy control iPSCs (Figure 5A,B). In particular, the high frequency of CP-iPSCs cells with more than five foci should be noted (Figure 5C). These foci colocalized with telomere sequences (Figure 5A).

**Figure 6:**
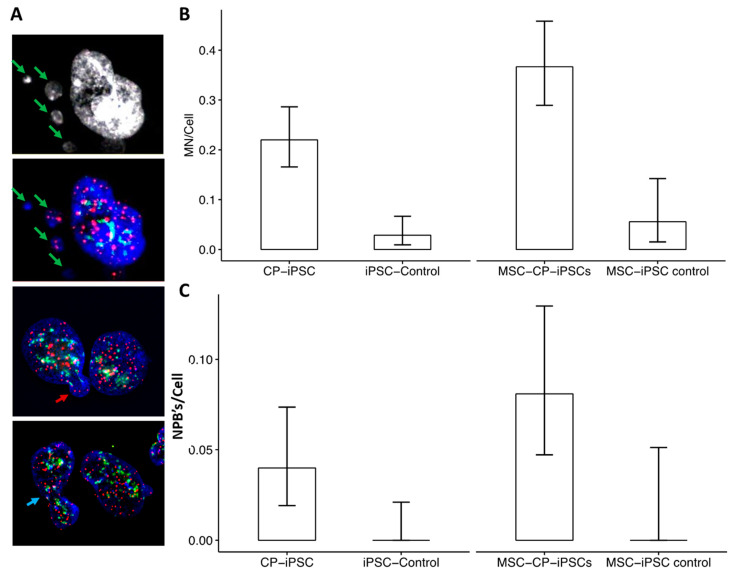
Chromosomal instability in CP-iPSCs and their derivative cells (**A**) Telomere (red) and centromere (green) signals reveal the presence of micronuclei (MN) (green arrow) with only telomere sequences related to the lagging acentric chromosome, nuclear buds (NBUD) (red arrow), and anaphase bridge formation (NPBs) (blue arrow) in CP-iPSCs. (**B**) Significant difference in micronucleus rate between CP-iPSCs and control iPSCs, as well as in derivative cells. (**C**) NPBs detected in CP-iPSCs and derivative cells, as well as in control iPSCs. No NPBs were detected in control iPSCs; the presence of NPBs was specific to CP-iPSCs and their derivative MSCs.

These markers of double-strand breaks and activation of the DNA repair machinery were associated with a higher frequency of micronucleus formation (Figure 6A) and anaphase bridges in CP-iPSCs and in their derived cells than in cells of healthy donors. Moreover, these aberrations could be related to the presence of dicentric chromosomes, a driving force of chromosomal instability in CP-iPSCs (Figure 6A,B). 

**Figure 7:**
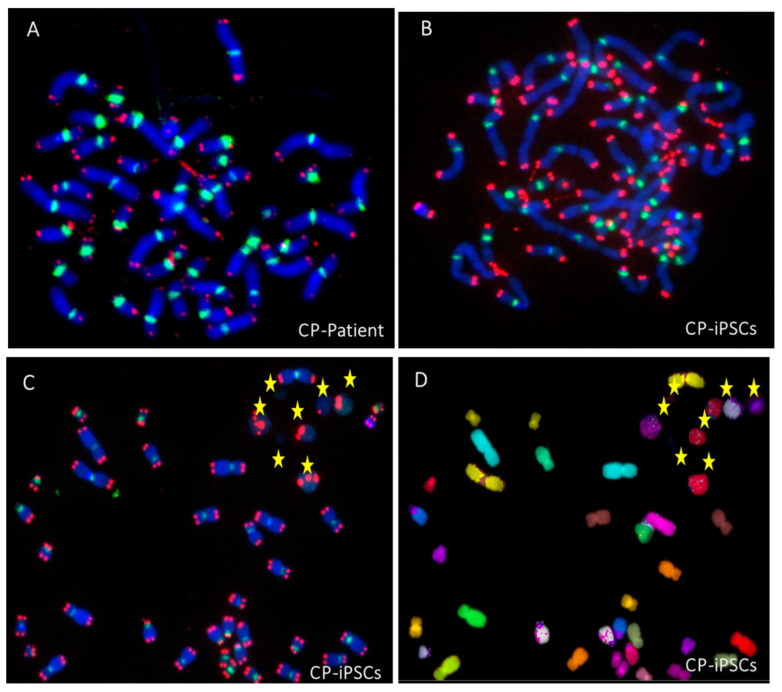
Telomere instability leading to chromosomal instability in Coats syndrome. (**A**) High rate of telomere aberrations in circulating lymphocytes of CP patient associated with the presence of telomere fusion and the formation of dicentric chromosomes after telomere (red signal) and centromere (green signal) staining. (**B**) Telomere fusions were detected in iPSCs from late passages and associated to the presence of acentric chromosomes. (**C**) The presence of micronuclei with only telomere sequences (yellow star) related to the formation of these acentric chromosomes. (**D**) M-FISH technique (each chromosome with a specific color) confirming the origin of these acentric chromosomes (yellow star) (63× magnification for all images).

Taken together, these results suggest that telomere dysfunction is a prominent feature of CP-iPSCs, although the degree of telomere dysfunction appears to vary by the number of passages of CP-iPSCs and iPSC-derived MSCs. This telomere dysfunction was similar to that observed in circulating lymphocytes of the CP patient, leading to chromosomal instability by breakage/fusion/bridge (B/F/B) cycles (Figure 7A,B). Higher rates of acentric chromosomes and micronuclei were observed in CP-iPSCs than in control iPSCs (Figure 7B–D).

### 3.5. CP-iPSCs Exhibit Features of Cellular Senescence and Increased Radiosensitivity

**Figure 8:**
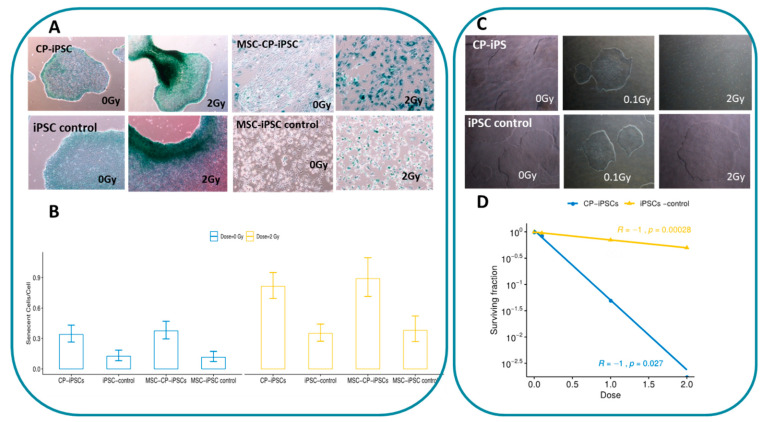
Response of CP-iPSCs cells to genotoxic agents. (**A**) Basal and induced senescent cells detected by staining for the senescence marker β-Galactosidase. (**B**) Significantly higher rate of senescent cells in CP-iPSC cells compared to iPSC control before and after irradiation. Similar results were obtained after the analysis of MCS derivative cells. (**C**) iPSC proliferation after radiation doses of 0.1 and 2 Gy. (**D**) Surviving fraction of CP-IPSCs and control iPSCs after exposure to radiation at 2 Gy.

To assess the relationship between telomere dysfunction and the senescence phenotype of CP cells, the senescence marker SA-βgal was analyzed in CP-iPSCs and MSCs and compared to control iPSCs before and after a dose of radiation of 2 Gy. One week after exposure, senescence was scored in cultures of nonirradiated and irradiated cells. Irrespective of irradiation, a significantly higher frequency of senescent cells was observed in CP-iPSC cultures compared to those in iPSCs from healthy donors, as well as MSC-CP-iPSCs (Figure 8A,B).

**Figure 9:**
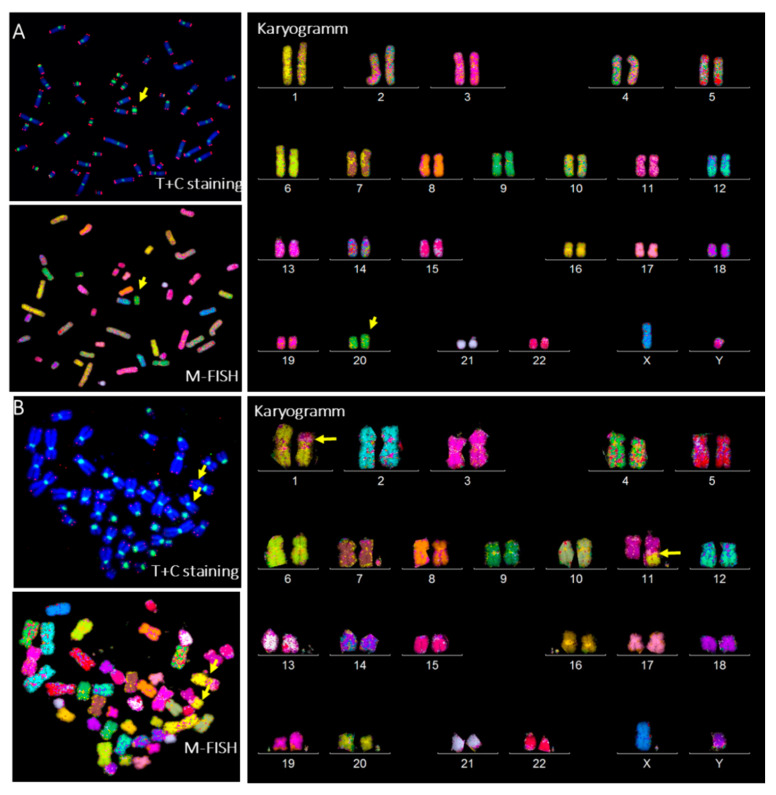
Nature of induced of chromosomal instability in CP-iPSC cells. (**A**) Representative image of metaphase of CP-iPSCs after telomere and centromere following M-FISH staining and karyotype of CP-iPSCs showing the presence of a clonal dicentric chromosome (iso(20q)) (yellow arrow). (**B**) Metaphase from healthy control iPSCs irradiated similarly to CP-iPSCs, demonstrating the presence of a reciprocal translocation t (1;11) (p21;q21) (yellow arow) (63× magnification).

Next, we studied the radiation sensitivity of CP cells. The surviving fraction of irradiated cells after exposure to different doses of irradiation was analyzed. CP-iPSCs did not proliferate after 1 Gy (Figure 9B), and no surviving fraction was observed at 2 Gy (SF2~0).

Three weeks after exposure to a low dose (0.1 Gy) of irradiation, the surviving CP-iPSCs were irradiated again, but at a high dose (4 Gy). A high rate of cell survival was observed in this condition, with this rate being similar to that observed in control iPSC cells leading to higher radiation resistance and modification in terms of response to the ionizing radiation of CP-iPSCs cells.

### 3.6. Radiation-Induced Chromosome Instability and Formation of Clonal Dicentric Chromosomes in CP-iPSCs

After in vitro radiation at the low dose of 0.1 Gy and subsequently at 4 Gy, radiation-induced clonal dicentric chromosomes were identified in all CP-iPSCs metaphases. Using sequential analysis with the TC + M-FISH technique, we detected a recurrent pattern of clonally expanding chromosomal rearrangements, characterized by an iso-chromosome 20 (Figure 9A). No interstitial telomere signals were seen in these aberrations. This suggests that the destabilization of chromosome 20 caused by telomere loss or deletion was stably fixed in mitosis by duplication of the q arm of chromosome 20 and the formation of an iso-20q. Of note, higher frequencies of anaphase bridges and micronuclei were observed in CP-iPSCs after irradiation. No dicentric chromosomes were identified in healthy control iPSCs after irradiation using the same protocol. The frequency of chromosomal aberrations induced by irradiation is depicted in Figure 9B.

## 4. Discussion

Telomeres play a key role in the maintenance of genome stability and integrity. Telomere dysfunction is one of the hallmarks of aging and cellular senescence [30,31]. The detrimental effects of accelerated telomere dysfunction, leading to chromosomal instability and organ failure, are well documented [19]. In recent years, an increasing interest [32] in the implications of telomere gene mutations in genetic disorders, called telomeropathies, has gained insight into the pathophysiology of hematological malignancies, specifically hematopoietic stem-cell disorders with bone marrow failure, as well as in disorders of premature aging [33].

The CP syndrome is a complex disorder with multiple manifestations [7]. Previous studies have demonstrated that mutations in *CTC1* are causal in the CP pathophysiology and lead to deficient telomere maintenance [34]. Molecular consequences of *CTC1* mutations have been reported previously [9], but telomere dysfunction and the telomere maintenance mechanisms related to the CP phenotype are still not well understood [11]. Moreover, *CTC1* mutations have also been reported in other diseases, underscoring the genotype–phenotype complexity of these telomere-related genetic disorders [35,36].

Several experimental models have been employed to address the role of *CTC1* mutations in telomere dysfunction [9,11,12,37]. However, these models provide limited molecular data to explain a correlative nature of most clinical observations. 

In the present study, we first showed the feasibility of generating iPSC lines from a patient with a severe Coats plus syndrome caused by deleterious mutations in both *CTC1* alleles. Telomerase and classical markers of pluripotency were expressed constitutively in these iPSCs. Furthermore, the cells could differentiate into various cell lineages by culture under appropriate conditions. Thus, our iPSCs present a model system to study telomere dysfunction of CP syndrome, as *CTC1* plays a major role in telomere maintenance as part of the heterotrimer CST complex. Importantly, our data demonstrate that, in contrast to the patient’s peripheral blood lymphocytes which exhibit short telomeres, the lengths of telomeres were increased after reprogramming into iPSCs. However, during their successive passages, telomeres became progressively shortened, suggesting a deficiency in the telomere maintenance mechanisms. 

In peripheral blood lymphocytes of the CP patient, telomeres were significantly shorter than in those of healthy donors with similar age. However, in contrast to lymphocytes from patients with other syndromes with telomere gene mutations, no drastic telomere shortening was observed in cells of this CP-patient [12]. Although extensive telomere shortening is not considered a general hallmark of the CP syndrome [6,34,36,38], the cells of the present patient displayed abundant telomere length heterogeneity with both very short and very long telomeres. This heterogeneity correlated with conspicuous and irregular morphologies of the nuclei. Thus, in this context, we propose that protein expression involved in maintenance of the nuclear membrane such as lamin B1 should be investigated in this disease [39,40]. In the cytogenetic analyses of the patient’s peripheral blood lymphocytes, we detected high frequencies of telomere loss and deletions related to telomere dysfunction. These observations led us to screen metaphase preparations for the presence of dicentric chromosomes, which are gold-standard markers of chromosomal instability in the absence of irradiation. Using in situ hybridization with probes specific for telomere and centromere sequences, we detected a high frequency of dicentric chromosomes in circulating lymphocytes of the CP patient compared to those from healthy controls. These aberrations occurred concomitantly with breakage/fusion/bridge (B/F/B) cycles. B/F/B cycles are initiated when sister chromatids fuse related to telomere loss, form a bridge during anaphase, and then break when the centromeres are pulled to opposite poles during anaphase [18]. Thus, this work demonstrates, for the first time, that telomere dysfunction in a Coats plus syndrome patient resulted in chromosomal instability manifested by the formation of dicentric chromosomes [41]. The irregular cellular phenotype could be caused by premature senescence related to the *CTC1* mutations.

Previous studies have established that reprogramming of human [42] and murine [43] somatic cells into pluripotency is associated with activation of telomerase expression and telomere elongation. In CP-iPSCs, telomeres were elongated with a higher rate of telomerase expression compared to healthy iPSCs. This finding suggests that the aberrant CTC1 proteins cause malfunction of the CST complex and, thus, upregulation of telomerase expression and telomere elongation. Concomitantly, the ALT mechanism for maintenance of telomeres was activated in CP-iPSCs. The presence of both mechanisms of the telomere maintenance in CP-iPSCs may be explained by the complexity of the disease per se, by the *CTC1* variants, and/or by the existence of additional unknown mutations in this syndrome. It should be mentioned that telomere maintenance mechanisms in iPSC cells derived from patients with different telomeropathies such as aplastic anemia do not exhibit a similar profile [44].

Nevertheless, enhanced telomere shortening following multiple passages was more pronounced in CP-iPSCs compared to iPSCs from healthy donors and associated with an increase in telomere length heterogeneity. In late passages, telomere aberrations were also associated with irregular changes of nuclear morphology. These events led to the progression of telomere dysfunction after the reprogramming of iPSCs cells. Interestingly, telomere dysfunction was more pronounced in derivative cells such as mesenchymal stem cells.

The second important characteristic of the CP syndrome is chromosomal instability related to telomere dysfunction. In CP-iPSCs cells, higher rates of micronuclei and anaphase bridges were detected, reflecting a similar profile to that in circulating lymphocytes of the CP patient. A close correlation between telomere dysfunction and chromosomal instability was demonstrated previously [15,45]. Using the iPSC model, we demonstrated that the progression of formation of telomere aberrations correlated with enhanced telomere shortening and chromosomal instability via B/F/B cycles in the CP syndrome. The similarity of these results with those obtained using circulating lymphocytes of the CP patient demonstrates that our iPSC model mirrors the telomere pathology of this disease.

The third important finding in this study is the enhanced radiation sensitivity and profile of senescent CP-iPSCs. These cells and their derivatives showed a higher frequency of senescent cells in both nonirradiated and irradiated cultures compared to cultures of iPSCs of healthy donors. Poor tolerance to chemo/radiation therapy, as well as bone marrow failure requiring stem-cell transplantation in patients with telomeropathies have previously been described [46,47,48,49]. In CP-iPSCs cells, radiation sensitivity was associated with telomere dysfunction and foci of DNA damage proteins, concomitantly with senescent cells. The formation of clonal dicentric chromosome in CP-iPSCs cells after irradiation could illustrate this dysfunction, as well as progressing genomic instability, in this disease [25].

## 5. Conclusions

To our knowledge, this is the first report on the establishment of an iPSC line derived from a compound heterozygotic patient with severe Coats plus syndrome caused by deleterious mutations in *CTC1*. We investigated correlations among loss of telomere function, chromosome instability, and enhanced radiation sensitivity using somatic cells and iPSCs from healthy donors, as well as *CTC1*-mutated somatic cells and iPSCs from a CP patient. CP-iPSCs cells showed similar features to those of the patient’s primary somatic cells in terms of telomere dysfunction and chromosomal instability. We anticipate that our iPSC-based disease model will advance our understanding of the molecular biology of the CP syndrome and will be a valuable tool for investigating putative therapeutics for patients with this devastating disorder.
